# Mandibular undifferentiated pleomorphic sarcoma: Molecular analysis of a primary cell population

**DOI:** 10.1002/cre2.301

**Published:** 2020-07-11

**Authors:** Hope M. Amm, Patricia DeVilliers, Ambika R. Srivastava, Marina G. Diniz, Gene P. Siegal, Mary MacDougall

**Affiliations:** ^1^ Oral and Maxillofacial Surgery University of Alabama at Birmingham Birmingham Alabama USA; ^2^ Department of Pathology University of Alabama at Birmingham Birmingham Alabama USA; ^3^ Department of Biomedical Engineering University of Alabama at Birmingham Birmingham Alabama USA; ^4^ Department of Pathology and Oral Surgery and Pathology Universidade Federal de Minas Gerais Belo Horizonte Brazil; ^5^ Department of Genetics University of Alabama at Birmingham Birmingham Alabama USA; ^6^ Faculty of Dentistry The University of British Columbia Vancouver British Columbia Canada

**Keywords:** MMPs, NOTCH, oral cancer, primary cells, sarcoma

## Abstract

**Background:**

Undifferentiated pleomorphic sarcomas are one of the most common subtypes of soft tissue sarcomas. These are aggressive mesenchymal tumors and are devoid of the major known biomarkers except vimentin. Our objective was to establish and characterize a primary cell population from a mandibular UPS specimen.

**Methods:**

The tumor was surgically removed from the right mandible of a 24‐year‐old male with IRB approved signed consent. Tumor was dissected, cultured ex vivo, and a cell population, MUPS‐1, were isolated from outgrowths. Gene and protein expression profiles of both the primary tumor and the derived there from cells were obtained by quantitative RT‐PCR and immunohistochemistry and included markers of epithelial, endothelial, and mesenchymal differentiation. To better define potential biomarkers, MUPS‐1 cells were additionally characterized by RNA sequencing analysis.

**Results:**

Pathological analysis of primary tumor tissue revealed a sarcoma demonstrating multiple pathways of differentiation simultaneously with myxoid, fibrous, and osseous tissue. The isolated cells had a spindle cell‐like morphology, were maintained in culture for greater than 20 passages, and formed colonies in soft agar indicating tumorigenicity. The cells, similar to the primary tumor, were strongly positive for vimentin and moderately expressed alkaline phosphatase. RNA‐seq analysis revealed the tumor over‐expressed several genes compared to normal tissue, including components of the Notch signaling pathway, *NOTCH3* and *JAG1*.

**Conclusions:**

We have successfully established an undifferentiated pleomorphic sarcoma cell population, which will provide a valuable resource for studying fundamental processes and potentially serving as a platform for exploring therapeutic strategies for sarcomas.

## 1 INTRODUCTION

Soft tissue sarcomas (STS) encompass a diverse group of tumors arising from mesenchymal tissues such as cartilage, bone, fat, myxoid tissue, blood vessels, and muscle (Dodd, 2016; Matushansky et al., 2009; Tudor‐Green, Gomez, & Brennan, 2017). STS are separated into 80–100 subtypes based on histological and molecular phenoytpes (Jo & Fletcher, 2014; Salawu et al., 2016; Tudor‐Green et al., 2017). Undifferentiated pleomorphic sarcomas (UPS) are the most commonly diagnosed soft tissue sarcoma in adults; however, this should not be used as a diagnosis of exclusion. Many histologic mimics began as seemingly better differentiated tumors of fat (liposarcoma), muscle (leiomyosarcoma), or other mesenchymal cell types such as bone and cartilage which over time give rise (de‐differentiate) into more aggressive, but morphologically undifferentiated tumors (Matushansky et al., 2009; Widemann & Italiano, 2018). Surgical resection is the primary treatment of UPS with some patients benefitting from the addition of chemotherapy. Even with surgical removal, the overall five‐year survival is 54.7%, local recurrence rates are 13–42%, and many patients develop metastatic disease (Fletcher, 2014; Roland et al., 2016). Adjuvant chemotherapy or radiation therapy may be recommended for later stage tumors.

Histologically, UPS is composed of highly cellular pleomorphic spindle cells with markedly atypical nuclear features and with a high mitotic index. UPS commonly occurs in the extremities and retroperitoneum of adult Caucasian males and are rarer in women and other racial and ethnic populations (Jo & Fletcher, 2014; Roland et al., 2016). They are uncommon in children and young adults; and the peak incidence is in the sixth and seventh decades of life. UPS tumors, such as the one described here, were previously occasionally defined as malignant mesenchymomas when these pluripotential tumors simultaneously demonstrated two or more malignant mesenchymal elements. Over time this entity has disappeared from use and is now incorporated under the umbrella of UPS.

UPS of the jaw (gnathic) are rare, expansible lesions, occurring the maxilla or mandible with few cases reported. In each case, the tumor was detected as a bone‐infiltrating mass. In this study, we describe a UPS extracted from the right mandible of a 24‐year‐old male. Pathological analysis of primary tumor tissue revealed a sarcoma demonstrating mixed morphologic patterns of the tissue with myxoid, fibrous, and osseous elements. This tissue was used to establish primary cell populations, which was characterized based on cell‐type specific markers and RNA sequencing.

## 2 MATERIALS AND METHODS

### 2.1 Tumor specimen and establishment of cell populations

Minced tumor pieces were collected following surgical resection of a right mandibular tumor following obtainment of informed consented under an institutionally approved IRB protocol. Part of the primary tumor was fixed in formalin and paraffin‐embedded. Remaining pieces were dissected, minced and placed in culture. Primary cell populations (MUPS‐1) were established as outgrowths in α‐DMEM/10%FBS/antibiotics.

### 2.2 Determination of cell growth rate

Cells were plated on a 96‐well plate and cell viability was measured using an MTS assay (Cell Titer96, Promega, Madison, WI) and absorbance at 490 nm (BioTek, Winooski, VT).

### 2.3 Soft agar colony formation assay

Soft agar colony formation was measured by seeding primary tumor cells, MUPS‐1, (2,500 cells) and positive control human breast cancer cells, MDA‐MB‐231 (1,500 cells), in medium containing 10% FBS and 0.3% agarose (UltraPure low melting point agarose, Invitrogen, Carlsbad, CA). Cells were plated over a layer of solidified medium containing FBS and 2% agarose in six‐well plates. Cultures were maintained in a humidified, 37°C incubator and media was changed every 3 days for 4 weeks before colonies were photographed and counted.

### 2.4 Quantitative real‐time PCR (qRT‐PCR)

Total RNA was isolated from cells using the Qiagen RNeasy Mini Kit (Valencia, CA). Formalin‐fixed paraffin‐embedded (FFPE) tumor sections (10 μm thick) were deparaffinized with xylene and ethanol (Steg et al., 2006). Tissue pellets were washed with ethanol and dried at 55°C. RNA was isolated using the Roche High Pure RNA paraffin kit (Roche Diagnostics, Indianapolis, IN). All RNA was converted to cDNA using the Bio‐Rad iScript cDNA Synthesis Kit (Herculus, CA). qRT‐PCR reactions were performed using the RT^2^SYBRGreen/Rox qPCR master mix (SABiosciences, Frederick, MD) and relative gene transcriptional levels detected using the ABI Prism 7,500 Sequence Detection System (Applied Biosystems, Carlsbad, CA). Primers for ameloblastin (*AMBN*), amelogenin (*AMELX*), amelotin (*AMTN*), CD34, cytokeratin 14 (*CK14*), enamelin (*ENAM*), hairy and enhancer of split 1 (*HES1*), hairy and enhancer of split 5 (*HES5*), hairy/enhancer‐of‐split related with YRPW motif 1 (*HEY1*), insulin‐like growth factor binding protein 3 (*IGFBP3*), jagged 1 (*JAG1*), jagged 2 (*JAG2*), kallikrein 4 (*KLK4*), matrix metalloproteinase‐3 (*MMP3*), *MMP12*, *MMP16*, *MMP20*, *NOTCH1*, *NOTCH2*, *NOTCH3*, *NOTCH4*, tissue inhibitors of metalloproteinase‐1 (*TIMP1*), *TIMP2*, *TIMP3*, and vimentin (*VIM*) were obtained from RT^2^ quantitative PCR primer assays (SABiosciences). Primers for *ACAN*, *ALP*, *BSP*, *DACT1*, *DMD*, *DSP*, *DSPP*, *HAPLN3*, *HEY2*, *HSPA1A*, *ID4*, *JPH2*, *MEPE*, *MMP1*, *MMP2*, *MMP11*, *MMP14*, *MMP17*, *MMP19*, *MMP23*, *MMP24*, *MMP28*, *OPN*, *RIPK4*, *S100A14*, and *WISP2* were synthesized (Invitrogen, Table S1). Cycle threshold (Ct) values corresponding to transcriptional levels were obtained and normalized to the GAPDH reference gene to calculate the delta Ct (dCt) value.

### 2.5 Next‐generation sequencing on Illumina platform

RNA samples were submitted to the Genomics Core Laboratory in the Heflin Center for Genomic Sciences at our institution for sample preparation and sequencing. mRNA‐sequencing was performed on the Illumina HiSeq2000. The quality of the total RNA was assessed using the Agilent 2,100 Bioanalyzer followed by two rounds of poly A+ selection and conversion to cDNA. TruSeq library generation kits were utilized as per the manufacturer's instructions (Illumina, San Diego, CA). The cDNA libraries were quantitated using qPCR in a Roche LightCycler 480 with the Kapa Biosystems kit for library quantitation (Kapa Biosystems, Woburn, MA) prior to cluster generation. Clusters were generated to yield approximately 725–825 K clusters/mm^2^. Cluster density and quality were determined during the run after the first base addition parameters were assessed. Paired end 2X50bp sequencing runs were performed to align the cDNA sequences to the reference genome.

### 2.6 RNA‐sequencing data analysis

TopHat version 2.0.9 (parameters: ‐r 150; −library‐type fr‐unstranded; −G; −transcriptome‐index) was used to align the raw RNA‐Seq fastq reads to the UCSC hg19 reference genome using the short read aligner Bowtie2 (1, 2, 3). TopHat also analyzes the mapping results to identify splice junctions between exons. Cufflinks version 2.2.0 (parameters: −g; −L; −b; −u) used the aligned reads from TopHat to assemble transcripts, estimate their abundances and test for differential expression and regulation (3, 4). In brief, correcting for sequence bias and using the upper‐quartile normalization options were turned on to better improve the transcript assembly and abundance estimates. Cuffmerge (parameters: −o; −g; −s), which is part of Cufflinks merged the assembled transcripts to a reference annotation and is capable of tracking Cufflinks transcripts across multiple experiments. Finally, Cuffdiff (parameters: −u; −library‐norm‐method geometric; −b; −L) identified significant changes in transcript expression, splicing and promoter use (Langmead, Trapnell, Pop, & Salzberg, 2009; Trapnell et al., 2010; Trapnell et al., 2012; Trapnell, Pachter, & Salzberg, 2009).

### 2.7 Immunohistochemistry

For immunohistochemistry (IHC) staining of the FFPE tissue, consecutive 5 μm thick sections were cut, deparaffinized and rehydrated. Citrate antigen retrieval was performed for 20 min at 97°C. Samples were incubated in hydrogen peroxide block (Thermo Scientific, Fremont, CA) for 15 min and Ultra V block for 5 min. For specific staining, samples were incubated with primary antibodies for 30 min and HRP polymer‐conjugated antibody for 15 min. Diaminobenzidine tetrachloride was used as the chromagen (Biocare Medical, Concord, CA) and hematoxylin for counterstaining. For immunocytochemistry, cells were grown in 4‐well chamber slides until 70% confluent then fixed with 4% formaldehyde. Staining was conducted as previously described above for tissue sections. Negative controls were incubated with PBST and secondary antibody without primary antibody. Samples were imaged with a Nikon Eclipse TE2000‐E inverted microscope (Nikon Instruments, Melville, NY).

Commercially available antibodies used were ALP, CK14, Jagged 1 (H‐66), OPN, and Notch3 (Santa Cruz Biotechnlogy, Santa Cruz, CA), pan‐keratin and VIM (Dako North America, Inc., Carpinteria, CA), WISP2 (Thermo Scientific, Rockford, IL), and CD34 (Ventana, Tuscan, AZ).

## 3 RESULTS

### 3.1 Clinical and pathological findings

**FIGURE 1:**
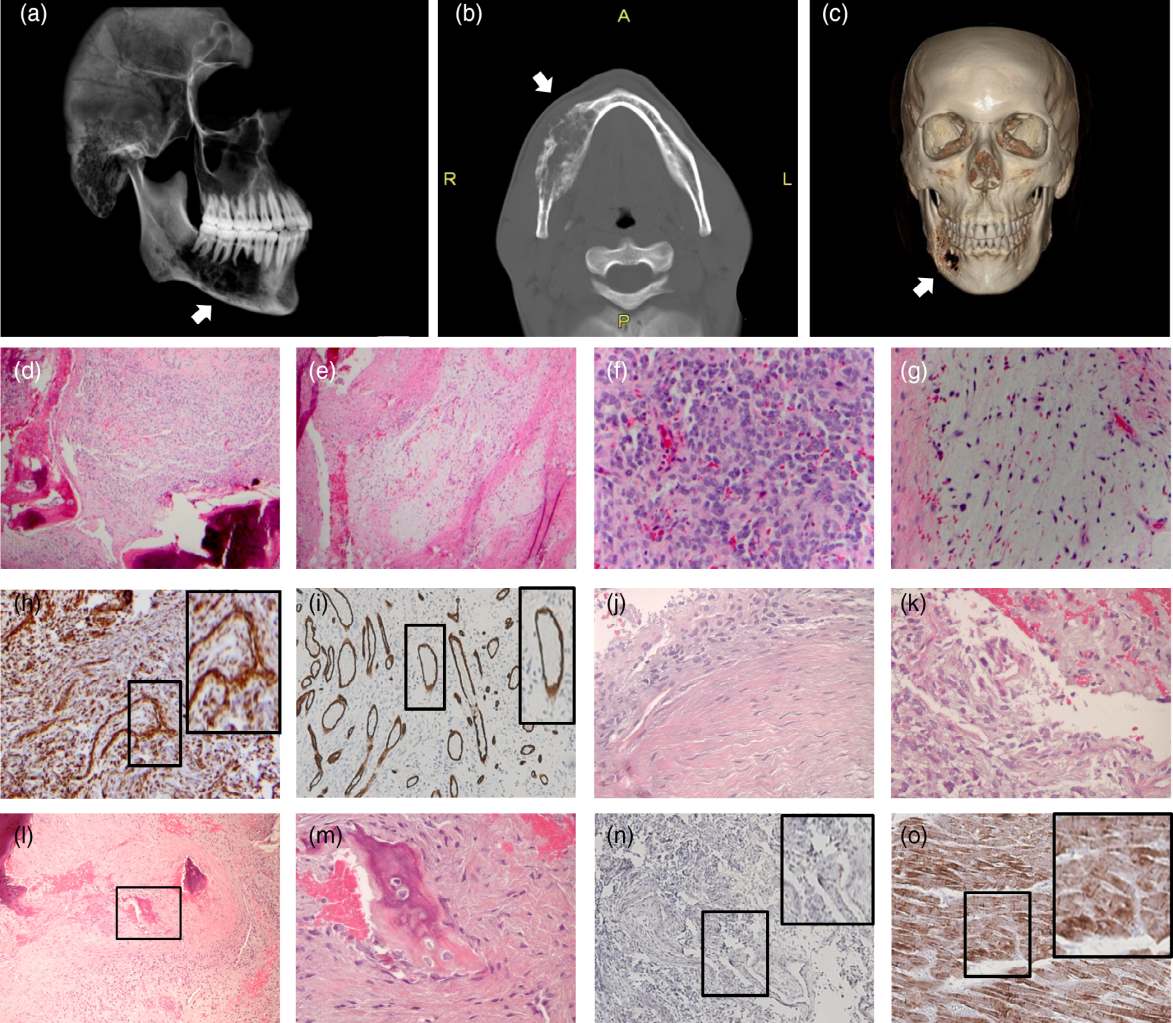
Clinical and pathologic examination of the undifferentiated pleomorphic sarcoma of the mandible. (a) Sagittal computed topography showing radiolucent lesion of the right mandible, later defined as undifferentiated pleomorphic sarcoma. (b) Axial plane computed topography of tumor lesion. (c) 3D reconstruction coronal view of the tumor lesion. (d–g) Hematoxylin and eosin (H&E) staining of tumor sections. Low (10x) and higher magifications (20x) as seen in panels (d and e), respectively. Round, pleomorphic tumor cells infiltrated with vascular channels (40x) are seen in panel (f) while panel (g) shows the spindle‐shaped tumor cells in a myxoid background and vascular channels (40x). (h) Strong positive staining for VIM, a mesenchymal tissue marker, in the tumor (20x). (i) CD34 staining demonstrating presence of vascular channels within the tumor (10x). (j–m). H&E staining of tumor sections showing cells of fibrous (j), myxoid (k), and osseous (l–m) histologic appearance. (n) Negative staining for desmin, indicating the tumor does not have striated muscle differentiation (10x). (o) Desmin positive control staining in human skeletal muscle (10x)

A 24‐year‐old African American male was seen with complaints of pain and swelling of his right mandible. A Panorex radiograph and facial CT scan showed an approximately 3x6 cm mixed radiopaque and radiolucent lesion with severe bone expansion and perforation at the right mandible with root resorption of the teeth around the right mandible (Figure 1a–c). Following biopsy, the patient was diagnosed with a sarcoma. The patient underwent neoadjuvant chemotherapy with four cycles of ifosamide and etoposide alternating with doxorubicin, isofamide, and vincristine followed by a right composite mandible resection and a left fibular free flap reconstruction. Post‐surgery he received a course of adjuvant therapy of ifosfamide and etoposide. He developed a postoperative wound infection, which required incision and drainage. A CT scan performed 2 years post‐surgery revealed no evidence of recurrence or metastatic disease (data not shown). He continued to resume normal activities and tolerates a regular diet.

The tumor was histologically characterized by an infiltrative proliferation of spindle and round cells in a background of numerous small vascular channels (Figure 1d–g) as highlighted by CD34 staining (Figure 1i). Cells of myxoid, fibrous, and osseous differentiation were identified among poorly differentiated tumor cells, and the tumor was diagnosed as an UPS (Figure 1j–m). The spindle cells from the biopsy specimen were negative (non‐reactive) for all markers tested (actin, smooth muscle actin, CD163, CD20, CD3, CD31, pan‐cytokeratin, desmin, MyoD1, myogenin), with the exception of VIM (Figure 1h–j). Absence of staining for desmin is shown as an example indicating the tumor does not have striated muscle differentiation (Figure 1n) while positive control staining is shown in human skeletal muscle tissue (Figure 1o).

### 3.2 Establishment and characterization of primary undifferentiated pleomorphic sarcoma cell population

**FIGURE 2:**
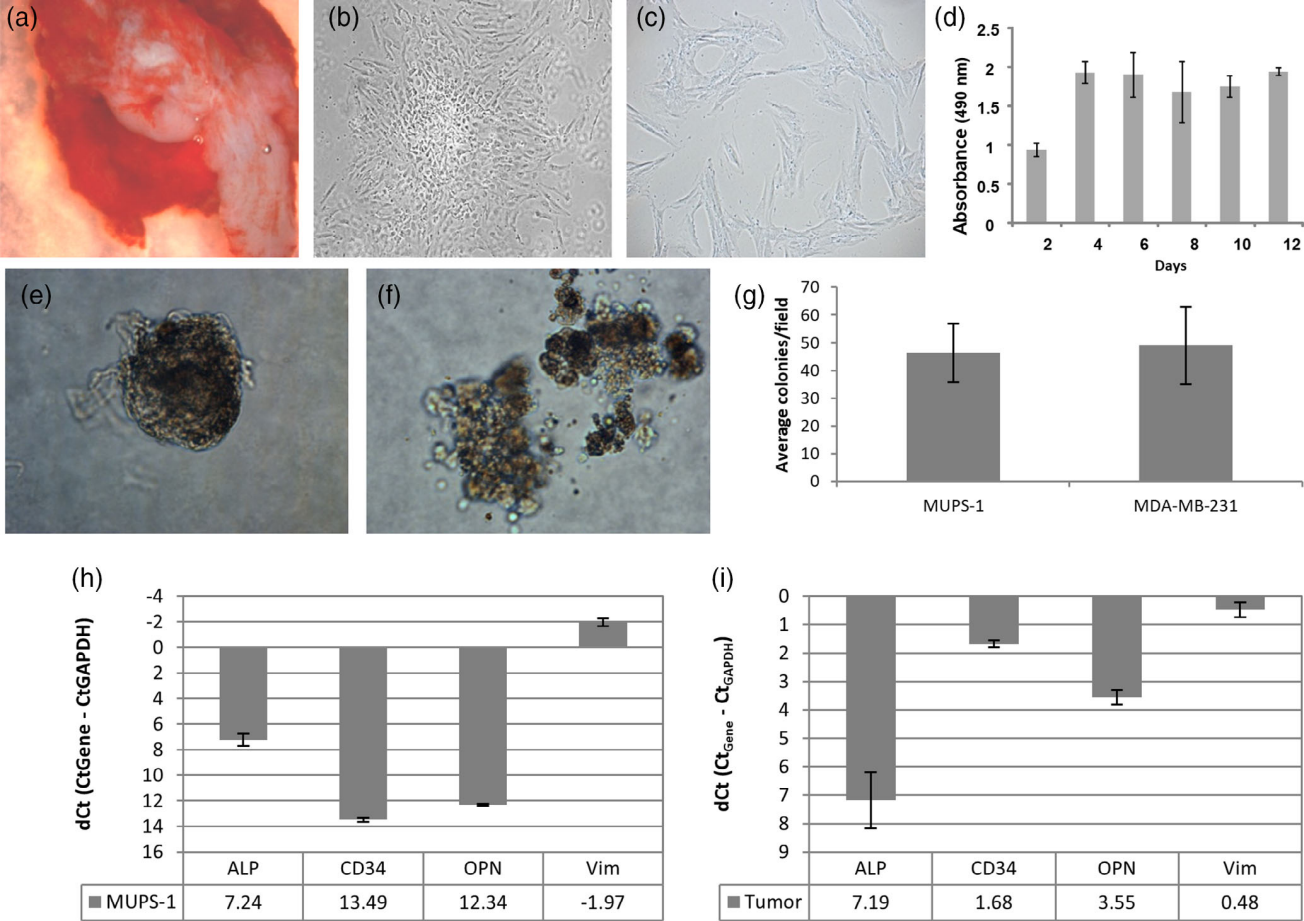
Establishment of the undifferentiated pleomorphic sarcoma (MUPS‐1) cell population. (a) Photomicrograph of primary tumor piece used for the ex vivo culture. Phase contrast micrograph of undifferentiated pleomorphic sarcoma cell population (MUPS‐1) at low (10x, b) and high (40x, c) magnification. (d) Growth rate of the MUPS‐1 cell population. (e) Soft agar colony formed by MUPS‐1 cells (40x). (f) Soft agar colony formed by positive control MDA‐MB‐231 cells (40x). (g) Quantification of colonies formed in soft agar assay as average number of colonies formed per three 4x fields. Gene expression profiles of cell type markers within the MUPS‐1 cell population (h) and primary tumor (i) were determined by qRT‐PCR. The 2 housekeeping gene GAPDH was used to normalize the dataset. All experiments were performed in triplicate and, and each reaction, in duplicate. The error bars indicate the SD

Surgically removed tumor pieces were dissected and used to establish primary cell populations for the purpose of characterization of the tumor and establishment of an in vitro cell model (Figure 2a). MUPS‐1 cells passaged from the initial tumor outgrowths had a spindle‐like appearance, a cell doubling time of 93.14 hr, organized into clusters when at confluency, and could be maintained in culture over 12 days (Figure 2b–d). Tumorigenicity of the MUPS‐1 cells was maintained in culture as measured by anchorage‐independent growth in soft agar. MUPS‐1 form colonies when suspended in agarose with complete media similar to the positive control MDA‐MB‐231 cells (Figure 2e–g).

**FIGURE 3:**
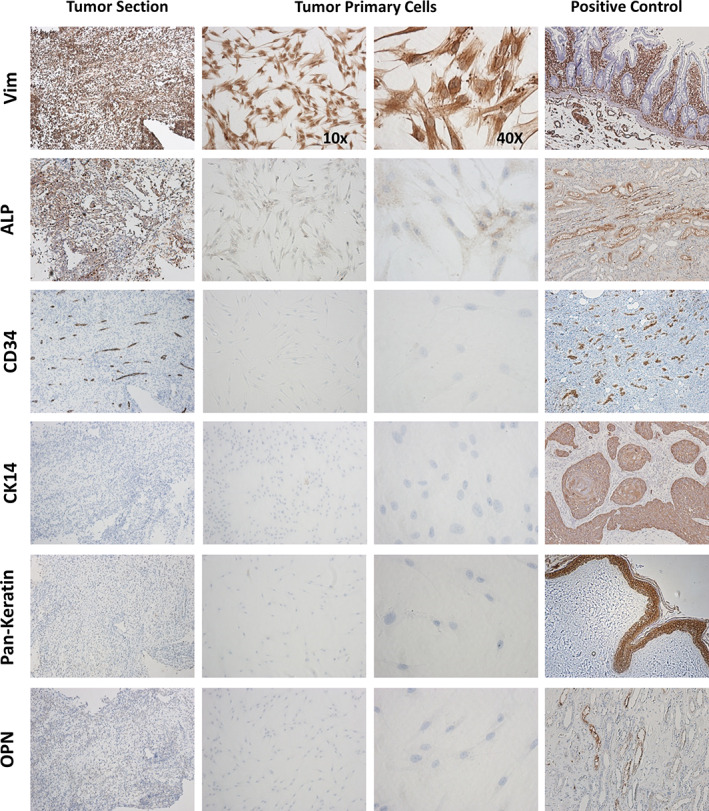
Immunohistochemical characterization of the primary undifferentiated pleomorphic sarcoma and the established MUPS‐1 cell population. Tumor and cells stained positive for VIM (Vim) and ALP, and negative for CK14, CD34, OPN, and pan‐keratin (tumor and controls, 10x; cells, 10x and 40x). Positively stained controls are provided for each antibody used in the experiment (10x)

For initial characterization of the cells, markers of various cell types were examined, including mesenchymal (*VIM*) and endothelial (*CD34*) cells (Figure 2h). At the transcript level (qRT‐PCR), the cells expressed high levels of *VIM* and low levels of *CD34* indicating the isolated cells were primarily mesenchymal in origin. The cells also expressed *ALP* and *OPN*, but not enamel matrix proteins (*AMELX*, *AMBN*, *AMTN*, *ENAM*), other bone and dentin SIBLING proteins (*BSP*, *DSPP*, *MEPE*) or the enamel proteinases *MMP20* and *KLK4* (data not shown). These results suggest that the isolated cells are not of odontogenic epithelium or mesenchymal dental pulp origin. To confirm origin of cells, RNA was isolated from the FFPE tumor. The original tumor expressed high levels of *VIM* similar to the isolated tumor cells (Figure 2i). Also expressed in primary tumor were *CD34* (the tumor was rich in blood vessels), and *OPN*, confirming the isolated cells express the same marker genes as the primary tumor. By immunohistochemistry, the cell populations showed strong positive staining with VIM (Figure 3). Sections of tumor tissue adjacent to the tissue used to establish the cell population was also strongly positive for VIM, similar to the original biopsy shown in Figure 1. The cells also expressed ALP, but less than 1% expressed CK14 and no protein expression of CD34 or OPN was detectable by IHC using commercial antibodies specific for these proteins (Figure 3).

**FIGURE 4:**
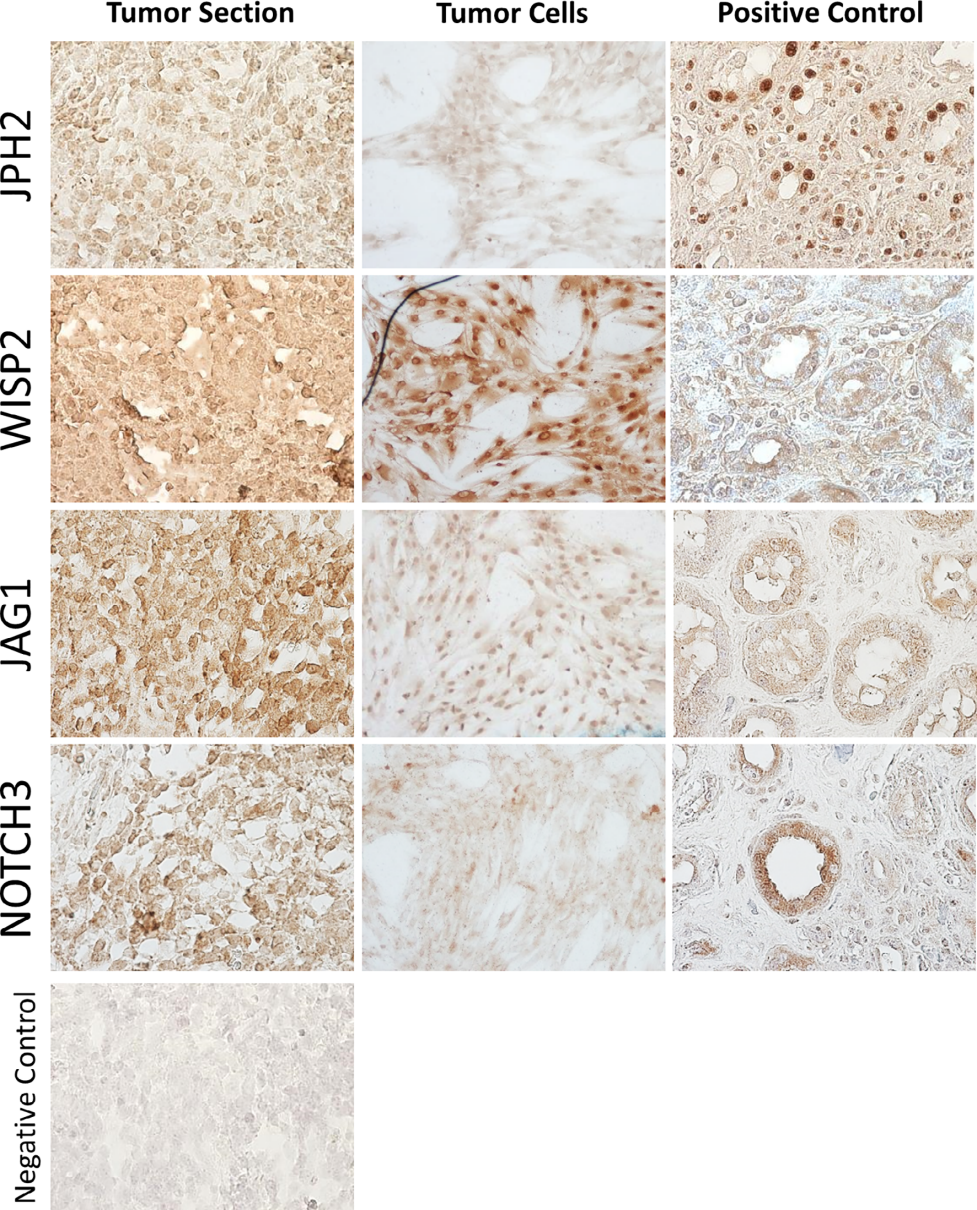
Immunohistochemical characterization of the primary undifferentiated pleomorphic sarcoma and the established MUPS‐1 cell population. Tumor and cells stained positive for JPH2, WISP2, JAG1, and NOTCH3 (10x). Positively stained controls are provided for each antibody used in the experiment (10x)

To better characterize the tumor cells and identify possible therapeutic targets, RNA sequencing analysis was conducted on RNA isolated from tumor cells and compared to dental pulp cells, which are of mesenchymal dental origin, similar to the MUPS‐1 cells. Thirty genes were upregulated in the MUPS‐1 cells compared to dental pulp cells, 11 of which were not expressed or expressed below the level of detection in p cells (Table 1). These genes were also among some of the most expressed in the MUPS‐1 cells. Fourteen genes were chosen based on previously reported relationships to human cancers and all 14 were confirmed to be upregulated in the MUPS‐1 cells by qRT‐PCR (Table 2). JPH2 and WISP2 proteins were both confirmed to be expressed in primary MUPS‐1 tissue and tumor cells (Figure 4). JPH2 may serve as a marker for UPS, as it was previously reported to be overexpressed in leiomyosarcomas compared to an endometrial stromal sarcoma (Davidson et al., 2013). Of particular interest, two genes associated with Notch signaling were upregulated in MUPS‐1 cells, a ligand for Notch, *JAG1*, and a receptor, *NOTCH3*. Both corresponding proteins were expressed in the primary tumor and MUPS‐1 cells suggesting the Notch pathway proteins may serve as markers for UPS.

**TABLE 1:** Genes differential expressed in MUPS‐1 cells compared to pulp cells

Gene symbol	Gene	MUPS‐1	ST‐001 pulp
ACAN	Aggrecan	137.65	0.00
AK4	Adenylate kinase 4	31.26	1.07
BGN	Biglycan	857.82	12.62
CABP1	Calcium binding protein 1	4.43	0.00
COL14A1	Collagen, type XIV, alpha 11	306.61	1.00
COL5A3	Collagen, type V, alpha 3	251.15	2.90
CRIP2	Cysteine‐rich protein 2	147.54	6.81
DACT1	Disheveled‐binding antagonist of beta‐catenin 1	84.15	1.62
DMD	Dystrophin	7.09	0.27
DSP	Desmoplakin	35.87	0.91
ELN	Elastin	269.05	0.75
FOXS1	Forkhead box S1	58.25	0.00
GGT5	Gamma‐glutamyltransferase 5	2.42	0.00
HAPLN3	Hyaluronan and proteoglycan link protein 3	128.53	4.39
HSPA1A	Heat shock 70 kDa protein 1A	2.23	0.00
ID4	Inhibitor of DNA binding 4	94.23	1.27
IGFBP3	Insulin‐like growth factor binding protein 3	186.00	6.90
JAG1	Jagged 1	189.37	3.30
JPH2	Junctophilin 2	37.74	0.96
KCNMB1	Potassium large conductance calcium‐activated channel	80.05	0.00
LPPR4	Plasticity‐related gene 1 protein	65.29	0.98
NOTCH3	Notch 3	279.62	7.56
PPP1R11	Protein phosphatase 1, regulatory (inhibitor) subunit 11	3.02	0.00
RIPK4	Receptor‐interacting serine–threonine kinase 4	6.10	0.00
S100A14	S100 calcium binding protein A14	2.46	0.00
SNORA75	Small nucleolar RNA, H/ACA box 75	4.06	0.00
TNMD	Tenomodulin, chondromodulin‐1‐like protein	7.61	0.00
TNS1	Tensin 1	48.67	1.99
TXNIP	Thioredoxin interacting protein	69.47	0.91
WISP2	WNT1 inducible signaling pathway protein 2	197.57	8.19

**TABLE 2:** qRT‐PCR Analysis of Selected Genes from RNA‐seq

Gene	Fold change
ACAN	15,117.38
DACT1	193.10
DMD	8.73
DSP	42.62
HAPLN3	109.50
HSPA1A	1.17
ID4	60.78
IGFBP3	9.91
**JAG1**	**945.64**
**JPH2**	**14.69**
**NOTCH3**	**51.74**
RIPK4	75.20
S100A14	3.60
**WISP2**	**238.00**

*Note*: Genes **bolded** were chosen from additional analysis.

To further investigate the role of Notch signaling in MUPS‐1 cells, additional ligands, receptors, and downstream transcriptional targets of Notch signaling were examined by qRT‐PCR. Notch transcriptional targets *HES1* (2.8 fold), *HES5* (195.7 fold), and *HEY2* (427.1 fold) were all upregulated in MUPS‐1 cells indicating active Notch signaling in these cells.

**FIGURE 5:**
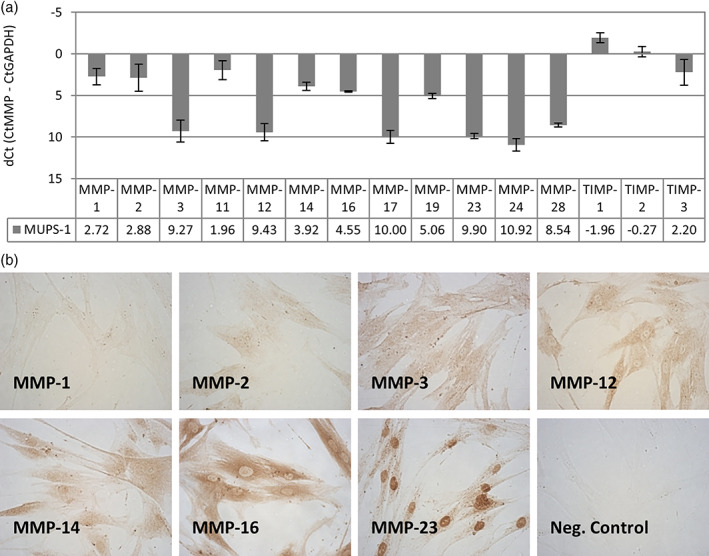
Matrix metalloproteinase (MMP) expression in MUPS‐1 cells. (a) Gene expression of MMPs in the MUPS‐1 cell population as determined by qRT‐PCR with GAPDH used to normalize the dataset. All experiments were performed in duplicate and repeated in triplicate, the error bars indicate S.D. (b) Immunohistochemical staining of MUPS‐1 cells with primary antibodies to MMP‐1, 2, 3, 12, 14, 16, and 23. Negative control is staining without primary antibody

This tumor presented intraosseously within the mandible demonstrating invasion and destruction of the jaw bone (Figure 1). The expression of MMPs in tumor cells was examined as MMPs are known to play a role in regulating the tumor microenvironment and promoting tumor invasion (Kessenbrock, Plaks, & Werb, 2010). The MUPS‐1 cells expressed several MMPs and TIMPs as detected by qRT‐PCR (Figure 5a). A subset of the expressed MMPs were examined by immunocytochemistry confirming expression at the protein level of MMP‐1, 2, 3, 12, 14, 16, and 23. The expression of MMPs was generally diffuse throughout the cells (Figure 5b). There was weak staining of MMP‐1 and 2. MMP‐16 had strong cytoplasmic staining, whereas MMP‐23 showed cytoplasmic as well as strong nuclear staining.

## 4 DISCUSSION

The establishment of primary UPS cell populations is useful in understanding the fundamental characteristics of this tumor and potentially providing a model for testing novel therapeutics. This is important considering UPS is a common subtype of soft tissue sarcoma with currently no clinical biomarkers available. Cellular and mouse models of UPS from various sites of the body have been developed (Becker et al., 2016; De Vita, Recine, Mercatali, et al., 2017; Kiyuna et al., 2017; Oyama et al., 2018; Salawu et al., 2016). The lower limbs, in particular the quadriceps muscle, was the most common tumor location. None were from the gnathic bones or from an intraosseous location. Few of these models were characterized beyond comparison to the primary tumor. De Vita et al. (2017) showed their UPS primary culture cells expressed increased *VIM*, *MMP2*, and *LAPTM4A* (a lysosomal transport gene) message compared to healthy tissue from the same patient. One study used UPS cells isolated from sporadic and radiation‐induced UPS via explant culture from primary tumors and passaged xenografts (May et al., 2017). UPS cell lines exhibited high levels of p‐Akt, the active form, which could be reduced with a dual PI3K/mTOR inhibitor. However, the PI3K/mTOR inhibitor increased phosphorylated insulin‐like growth factor receptor 1 (IGF1R), which is a known resistance mechanism to this inhibitor and was abrogated, in turn, with the addition of an anti‐IGF1R kinase inhibitor.

In the current study, we establish an UPS primary cell population that is mesenchymal in origin, as evident by positive VIM expression at the mRNA and protein level. The primary cells demonstrated tumorigenic capacity by anchorage‐independent growth and formation of colonies when suspended in medium‐enriched agar. The expression of mRNA and protein (VIM, ALP, CK14, CD34, OPN) in the MUPS‐1 cells matched the expression in the primary tumor confirming the origin of our cell population. The primary tumor and cells expressed high levels of VIM, as expected, and ALP, which has been shown to be expressed in mesenchymal stem cells (Gerlach et al., 2012).

Past studies to characterize molecular biomarkers of UPS demonstrated that UPS has a low burden of somatic mutations with 16% carrying *RB1* and/or *p53* mutations (Cancer Genome Atlas Research Network. Electronic address edsc, Cancer Genome Atlas Research N, 2017). One theory is that UPS arises from the transformation of mesenchymal stem cells (MSCs), which would account for the lack of differentiation seen in these tumors (Matushansky et al., 2009). Genetic analysis demonstrated that UPS cells showed more similarities with MSCs than cells from other sarcoma subtypes. Other studies have shown Ras/MAPK activation in 80% of UPS cases, and expression of phospho‐STAT3 associated with better prognosis (Bekki et al., 2017; Serrano et al., 2016). Another study showed that UPS and leiomyosarcomas had high expression of programmed cell death protein (PD‐1) and programmed death‐ligand1 (PD‐L1), and thus may benefit from treatment with immune checkpoint inhibitors (Pollack et al., 2017).

To characterize our cell model and identify possible biomarkers we preformed RNA‐sequencing analysis. Genes that were highly expressed in our UPS included *WISP2*, a regulator of Wnt signaling, which has been showed to play a role in the invasiveness of breast and colon cancer cells, and in epithelial‐to‐mesenchymal transition in pancreatic cancer (Davies, Watkins, Mansel, & Jiang, 2007; Dhar et al., 2007; Grunberg, Hammarstedt, Hedjazifar, & Smith, 2014). Notch3 and Jag1, components of the Notch signaling pathway, were increased in expression and confirmed by protein expression analysis in the primary tumor and tumor cells. Downstream Notch transcription factors were also overexpressed in MUPS‐1 cells (*HES1*, *HES5*, *HEY2*). Components of the Notch signaling pathway may thus potentially be used as biomarkers. In osteosarcoma, Notch signaling has been shown to play a role in invasiveness and resistance to chemotherapy (Ma et al., 2013; Tsuru et al., 2015; Wang, Wei, Han, et al., 2012). Notch inhibitors are currently being developed for the treatment of a variety of human malignancies and may be a viable option for the treatment of UPS when available.

The UPS tumor reported here had invaded and caused destruction within the mandible. The expression of MMPs has been associated with both remodeling of the tumor microenvironment in human tumors and tumor invasion in bone (Kessenbrock et al., 2010). These enzymes degrade extracellular matrices (ECM) allowing mobility of tumor cells as well as extravasation into blood vessels in the tumor microenvironment. Many MMPs are associated with human tumors and their increased expression has been correlated with poor prognosis (Westermarck & Kahari, 1999). MUPS‐1 cells expressed many MMPs. MMP‐2, expressed in our primary cells, may be related to the invasion of tumor cells and the development of the tumor vascular supply by allowing entry of endothelial cells during angiogenesis. Expression of MMP‐2 has shown to be correlated with poor prognosis in colon cancer patients and enhanced motility of tumor cells (Kryczka et al., 2012). De Vita et al. (2017) reported overexpression of *MMP2* in UPS cells. The expression of MMP‐2 in our primary tumor cells could be related to the highly vascular nature of the UPS demonstrated by CD34 staining of primary tumor. The activation of MMP‐2 is complex; MMP‐2 associates with TIMP‐2 and MMP‐14 to result in the proteolytic activation of MMP‐2 (Kessenbrock et al., 2010). The TIMP proteins are generally considered endogenous inhibitors of the MMP family; however, their role in MMP‐2 indicates an activator role of TIMP‐2. Additionally, expression of TIMPs has paradoxically been related to increased tumor aggressiveness and tumor cell proliferation (Murphy, 2011). MMP and TIMP expression correlated with the invasive nature of the UPS reported here.

Our study is limited by the enrollment and inclusion of one patient; however, we have extensively characterized the cells, including RNA‐sequencing analysis. A limitation of our analysis is the use of dental pulp cells as our control sample, which would share a dental mesenchymal origin, but the pulp cells would be differentiated. Our analysis identified novel potential biomarkers for UPS and STS, and with further investigation could be useful in pathologic studies. In addition, analysis linked a known tumor‐associated pathway with STS. The Notch pathway has been widely studied and may provide possible targeted therapies.

An advantage of our study is there are currently few commercially available sarcoma cell lines for preclinical studies, which are necessary for advancing therapies for UPS and STS. Miserocchi et al. (2017) highlight the advantages of ex vivo culture strategies such as the one employed here. These models avoid some of the limitations of traditional cultured cell lines, which are generally clonal selected producing a homogenous cell population not represent of the tumor microenvironment. ex vivo primary cultures are not clonally selected or manipulated to be immortal allowing the maintenance of tumor heterogeneity, which may play roles in drug resistance and tumor recurrence. The ability to grow cells in 3D conditions has also proven to more closely match the morphology, growth, and cell–cell connections of the original patient tumor compared to monolayer cultures (Hudson, Fox, Luckett, & Manson, 2006; Miserocchi et al., 2017). The cells MUPS‐1 cells described here were shown to have the capacity to grow in a 3D matrix (Figure 2e) and future studies could employ this model.

In summary, we established a cell population, MUPS‐1, from a gnathic UPS. We characterized the expression of mesenchymal cell markers and demonstrated a lack of dental cell markers and epithelial markers in the isolated primary cells and the tumor. We characterized the tumor based on RNA sequencing and confirmed results showing protein expression in the primary tumor and MUPS‐1 cells. Among novel biomarkers identified were JPH2, WISP2, and components of the Notch pathway. The expression of MMPs may be regulating the extensive bone invasion and vascularization of the tumor. The development of primary cell populations provides a valuable resource for characterizing human tumors, and allows for preclinical studies for the development of novel therapeutics.

## CONFLICT OF INTEREST

Supported by NIDCRT90‐DE022736‐01(DART), NIDCRK99/R00‐DE023826, the Haley's Hope Memorial Support Fund, and the Thomas Logan RAID Fund at UAB.

## Supporting information


**Table S1.** Quantitative real‐time PCR primers.
