# Supplementary material for: Mandibular undifferentiated pleomorphic sarcoma: Molecular analysis of a primary cell population
Source: Clin Exp Dent Res. 2020 Jul 11;6(5):495–505. doi: 10.1002/cre2.301 (PMC7545231; doi:10.1002/cre2.301)
Supplement: Supplementary file 1 — Table S1. Quantitative real‐time PCR primers. [file CRE2-6-495-s001.docx]

Supplemental Table 1. Quantitative Real-Time PCR primers.

| **Gene** | **Name** | **Primer Sequence** | **Reference** |
| --- | --- | --- | --- |
| ACAN | aggrecan | F 5'-GCAGCTGGGCGTTGTCA-3' R 5'-TGAGTACAGGAGGCTTGAGGACT-3' | N/A |
| ALP | alkaline phosphatase | F 5’-ATGGGATGGGTGTCTCCACA-3’  R 5’-CCACGAAGGGGAACT TGTC-3’ | N/A |
| BSP | bone sialoprotein | F 5'-TGCCTTGAGCCTGCTTCC-3’  R 5'-AAAATTAAAGCAGTCTTCATTTTG-3' | D’Souza 2011 |
| DACT1 | dishevelled-binding antagonist of beta-catenin 1 | F 5'-CACAAGCGAACTGACTACCG-3' R 5'-GTAATTGCTCTGCTCGTCCT-3' | Yuan 2012 |
| DMD | dystrophin | F 5'-TCAACAGGATTTTGTGACCAGC-3' R 5'-GCAACTTCACCCAACTGTCTTG-3' | N/A |
| DSP | desmoplakin | F 5'-GCTTGCCAACTTCAGAGGTTCT-3' R 5'-TTGGAGAATAGCCTGGAGCAGT-3' | Yang 2012 |
| DSPP | dentin sialophosphoprotein | F 5’-GAAGATGCTGGCCTGGATAA-3’  R 5’-TCTTCTTTCCCATGGTCCTG-3’ | Nam 2011 |
| HAPLN3 | hyaluronan and proteoglycan link protein 3 | F 5'-ATGAAAGCGGTCTGGTGGAG-3' R 5'-GGCCCTCGTGGAAGTTGAA-3' | N/A |
| HEY2 | hairy/enhancer-of-split related with YRPW motif protein 2 | F 5'-TTGTCAGTATCAGCCACGTC-3' R 5'-AGTTACCGAGCTGCCTTGAA-3' | Jeon 2014 |
| HSPA1A | heat shock 70kDa protein 1A | F 5'-AATCTTGGAAAGGCCCCTAA-3' R 5'-GAGCAGGTGTGTAACCCCAT-3' | N/A |
| ID4 | inhibitor of DNA binding 4 | F 5'-GTGCGATATGAACGACTGCT-3' R 5'-CAGGATCTCCACTTTGCTGA-3' | Jeon 2014 |
| JPH2 | junctophilin 2 | F 5'-TGCCTCAGCTTTGGCCT-3' R 5'-GCCAGAAAGTGGAGCACAGT-3' | Davidson 2013 |
| MEPE | matrix extracellular phosphoglycoprotein | F 5’-TGGCCTGAGGATGTCAAT TTATC-3’  R 5’-GAGAGCTGCGCCATATTCTTC-3’ | N/A |
| MMP-1 | matrix metalloproteinase-1 | F: 5’-GACAGAAAGAGACAGGAGAC-3’  R: 5’-GAGTTATCCCTTGCCTATCC-3’ | Hegedus 2008 |
| MMP-2 | matrix metalloproteinase-2 | F 5’-GCTGGCTGCCTTAGAACCTTTC-3’  R 5’-GAACCATCACTATGTGGGCTGAGA-3’ | Hegedus 2008 |
| MMP-11 | matrix metalloproteinase-11 | F 5’-CAACATACCTCAATCCTGTCCC-3’  R 5’-CAATGGCTTTGGAGGATAGC-3’ | Hegedus 2008 |
| MMP-14 | matrix metalloproteinase-14 | F 5’-GAGCTCAGGGCAGTGGATAG-3’  R 5’-GGTAGCCCGGTTCTACCTTC-3’ | Hegedus 2008 |
| MMP-17 | matrix metalloproteinase-17 | F 5’-ACTCATGTACTACGCCCTCA-3’  R 5’-GAGAAGTCGATCTGGATGTC-3’ | Hegedus 2008 |
| MMP-19 | matrix metalloproteinase-19 | F 5’-GGGTCCTGTTCTTCCTACAT-3’  R 5’-CAATCCTGCAGTACTGGTCT-3’ | Hegedus 2008 |
| MMP-23 | matrix metalloproteinase-23 | F 5’-CCAGAAGATCCTCCACAAGA-3’  R 5’-CAGGTGTAGGTGCCCTCATT-3’ | Hegedus 2008 |
| MMP-24 | matrix metalloproteinase-24 | F 5’-GGCAAAAACACATCACCTAC-3’  R 5’-GGTCACTTTTGATCTCATGG-3’ | Hegedus 2008 |
| MMP-28 | matrix metalloproteinase-28 | F 5’-GAGGCATTCCTAGAGAAGTACGGA-3’  R 5’-CTGAAACGCTCTGATGGCATC-3’ | Hegedus 2008 |
| OPN | osteopontin | F 5'-CTGAAACCCACAGCCACA-3'  R 5'-TGTGGAATTCACGGCTGA-3' | Luo 2008 |
| RIPK4 | receptor-interacting serine-threonine kinase 4 | F 5'-CAGAAGAAGCCGTTTGCAGAT-3' R 5'-GAGGCGTATCAGGTGGCTG-3' | N/A |
| S100A14 | S100 calcium binding protein A14 | F 5'-ACTCGGGCAAAGAGGGTGA-3' R 5'-CTGGGCTGCTTATCTGGGAA-3' | Xu 2014 |
| WISP2 | WNT1 inducible signaling pathway protein 2 | F 5'-GCGACCAACTCCACGTCTG-3' R 5'-TCCCCTTCCCGATACAGGC-3' | N/A |

D'Souza S, del Prete D, Jin S, et al. Gfi1 expressed in bone marrow stromal cells is a novel osteoblast suppressor in patients with multiple myeloma bone disease. *Blood.* 2011;118(26):6871-6880.

Hegedus L, Cho H, Xie X, Eliceiri GL. Additional MDA-MB-231 breast cancer cell matrix

metalloproteinases promote invasiveness. *J Cell Physiol.* 2008;216(2):480-485.

Jeon H, Kim S, Jin X, et al. Crosstalk between glioma-initiating cells and endothelial cells drives tumor progression. *Cancer Res*. 2014;74(16):4482-4492.

Luo X, Chen J, Song WX, et al. Osteogenic BMPs promote tumor growth of human osteosarcomas that harbor differentiation defects. *Lab Invest.* 2008;88(12):1264-1277.

Nam S, Won JE, Kim CH, Kim HW. Odontogenic differentiation of human dental pulp stem cells stimulated by the calcium phosphate porous granules. *J Tissue Eng.* 2011;2011:812547.

Xu X, Su B, Xie C, et al. Sonic hedgehog-gli1 signaling pathway regulates the epithelial

mesenchymal transition (EMT) by mediating a new target gene, S100A4, in

pancreatic cancer cells. *PLoS One*. 2014;9(7):e96441.

Yuan G, Wang C, Ma C, et al. Oncogenic function of DACT1 in colon cancer through the

regulation of β-catenin. *PLoS One*. 2012;7(3):e34004.

Yang L, Chen Y, Cui T, et al. Desmoplakin acts as a tumor suppressor by inhibition of the

Wnt/β-catenin signaling pathway in human lung cancer. *Carcin*. 2012;33(10):1863-1870.
